# Susceptibility Testing of Bacteria Using Maldi-Tof Mass Spectrometry

**DOI:** 10.3389/fmicb.2018.01744

**Published:** 2018-08-06

**Authors:** Irene Burckhardt, Stefan Zimmermann

**Affiliations:** Department of Infectious Diseases, Medical Microbiology and Hygiene, University of Heidelberg, Heidelberg, Germany

**Keywords:** MALDI-TOF MS, susceptibility testing, bacteria, time to report, treatment

## Abstract

Matrix-assisted laser desorption ionization time-of-flight mass spectrometry (MALDI-TOF MS) was introduced into the microbiological routine more than 10 years ago. Since then it has almost replaced biochemical identification. It is unrivaled in terms of accuracy and cost. From a laboratory's perspective it would be an ideal method to replace classic susceptibility testing, that is Kirby-Baur agardiffusion or determination of minimal inhibitory concentrations (MICs). First reports on possible assays for susceptibility testing are more than 10 years old. However, the developments during the last 5 years were substantial. This review focuses with some exceptions on the progress, which was achieved during the last decade.

## Background and aim of the review: why do we need susceptibility testing for bacteria and why is time-to-result such an issue?

According to the World Health Report 2015 infectious diseases cause more than 30% of all deaths worldwide (WHO, 2015) and rapid and correct antibiotic therapy is the most important single factor for the survival of patients with bacterial infections (Kumar et al., 2009). However, initial therapy is based on the experience of the physician, his knowledge about the most common bacteria causing the disease and its statistically most common susceptibility profile. That means initial therapy is rapid but guided by experience from past cases not evidence from the actual case. The choice of empirical therapy can be terribly wrong. Ideally identification and susceptibility testing could be done shortly after the respective patient samples were taken. In reality bacteria have to be cultured on agar plates in laboratories specialized in medical microbiology before they can be identified and tested against antibiotics. In general this takes 1 day for growth and an additional day for identification and susceptibility testing. During the last 10 years we could witness the triumph of MALDI-TOF MS over biochemistry for bacterial identification from bacterial cultures (solid or liquid) (Clark et al., 2013; Patel, 2015; Singhal et al., 2015; Angeletti, 2016; Arca-Suárez et al., 2017; Tré-Hardy et al., 2017) or directly from patient material (Sandalakis et al., 2017). One of its main advantages is that identification using MALDI-TOF MS takes minutes. Identification using biochemistry necessitates growth of bacteria and often takes at least 1 day. However, the gold standard for susceptibility testing still is the determination of the minimal inhibitory concentrations (MICs) toward a selection of antibiotics. Again, growth of bacteria is essential and often takes at least 1 day. Accelerating susceptibility testing is the next big goal in medical microbiology and MALDI-TOF MS is the promising technology for achieving it. This review will focus on important technical developments and the progress toward rapid susceptibility testing using MALDI-TOF MS. Literature published until March 2018 was included into the review (see Table 1 for a list of references in alphabetical order and organism/resistance studied, spectrometer used, matrix used and range (m/z) studied).

**Table 1:** List of references in alphabetical order and organism/resistance studied, spectrometer used, matrix used and range (m/z) studied.

**References**	**Organism/resistance studied**	**Spectrometer**	**Matrix**	**Range studied (m/z)**
Bernardo et al., 2002	*S. aureus*/methicillin resistance	Reflex III MS^§^	sinapinic acid	1,000–10,000
Burckhardt and Zimmermann, 2011	gram-negative bacteria/carbapenem resistance	microflex LT^§^	HCCA^*^	440–530
Calderaro et al., 2017	Enterobacteriaceae/carbapenem resistance	microflex LT	DHB	100–1,200
Du et al., 2002	*S. aureus*/methicillin resistance	linear MALDI-TOF MS^$^	5-chloro-2 mercapto-benzothialzole	600–3,500
Edwards-Jones et al., 2000	*S. aureus*/methicillin resistance	Kompact MALDI 2 linear TOF MS^#^	5-chloro-2 mercapto-benzothialzole	500–10,000
Griffin et al., 2012	*E. faecium*/vancomycin resistance (*vanB*)	microflex LT	HCCA	2,000–20,000
Hrabák et al., 2011	gram-negative bacteria/carbapenem resistance	microflex LT	2,5-dihydroxybenzoid acid (DHB)	360–600
Idelevich et al., 2018	*K.pneumoniae, P. aeruginosa*, meropenem resistance	microflex LT	HCCA	2,000–20,000
Johansson et al., 2014a	*B. fragilis*/carbapenem resistance	microflex LT	HCCA	400–600
Johansson et al., 2014b	*B. fragilis*/carbapenem resistance	microflex LT	HCCA	400–600
Josten et al., 2014	*S. aureus*/methicillin resistance	Biflex II MS^§^ Vitek MS^&^	HCCA	n.a.
Jung et al., 2014a	P. aeruginosa/carbapenem resistance, aminoglycoside resistance, quinolone resistance	microflex LT	HCCA	2,000–10,000
Jung et al., 2016	gram-negative bacteria/beta-lactam resistance, aminoglycoside resistance, quinolone resistance	microflex LT	HCCA	2,000–20,000
Justesen et al., 2018	*B. fragilis*, clindamycin, meropenem, metronidazole resistance	microflex LT	HCCA	2,000–20,000
Lange et al., 2014	*Klebsiella* spp./carbapenem resistance	microflex LT	HCCA	2,000–20,000
Lau et al., 2014	*K. pneumoniae*/carbapenem resistance	microflex LT	HCCA	2,000–20,000
Nagy et al., 2011	*B. fragilis*/carbapenem resistance	microflex LT	HCCA	2,000–20,000
Nakano et al., 2014	*E. faecium*/vancomycin resistance (*vanA*)	microflex LT	HCCA	2,000–20,000
Oviaño et al., 2016	Enterobacteriaceae, *P. aeruginosa, A. baumannii*, carbapenem resistance		HCCA	300–600
Papagiannitsis et al., 2015	gram-negative bacteria/carbapenem resistance	microflex LT	2,5-dihydroxybenzoid acid (DHB)	360–600
Pardo et al., 2016	gram-negative bacteria/quinolone resistance	Vitek MS	HCCA	270–420
Pranada et al., 2016	*S. aureus*/methicillin resistance	microflex LT	HCCA	2,000–20,000
Ramos et al., 2016	Enterobacteriaceae, *P. aeruginosa, A. baumannii*, carbapenem resistance	Vitek MS	HCCA	400–600
Rotova et al., 2017	Enterobacteriaceae, *P. aeruginosa*, carbapenem resistance	microflex LT	HCCA, DHB	300–600
Sparbier et al., 2012	gram-negative bacteria/beta-lactam resistance (penicillins, cephalosporins, carbapenems)	microflex LT	HCCA	2,000–20,000
Sparbier et al., 2013	*S. aureus*/methicillin resistance	microflex LT	HCCA	2,000–20,000
Szabados et al., 2012	*S. aureus*/methicillin resistance	microflex LT	HCCA	2,000–20,000

§ *manufactured by Bruker Daltonic GmbH, Bremen, Germany*.

* *HCCA:α-cyano-4-hydroxycinnamic acid*.

$ *manufactured by Micromass, Waters Corporation, USA*.

& *manufactured by bioMérieux, Nürtingen, Germany*.

# *manufactured by Kratos Analytical, Shimadzu Corporation, Japan*.

*n.a.: not available*.

## Assays using defined marker peaks to deduce susceptibility or resistance (single peaks, cluster of peaks, whole spectra)

### Methicillin-resistance-MRSA

At the turn of the millennium the most famous and most feared bacterium was methicillin-resistant *Staphylococcus aureus* (MRSA). Therefore, it is not surprising that the first attempts to use MALDI-TOF MS for susceptibility testing were made with *S. aureus*. The aim was to determine whether a specific *S. aureus* isolate was susceptible or resistant toward methicillin, i.e., MSSA or MRSA. As early as 2000 Edwards-Jones and colleagues (Edwards-Jones et al., 2000) studied 20 different staphylococcal strains: 7 MSSA (all reference strains from official strain collections), 7 MRSA (clinical isolates only) and 6 non-*S. aureus* staphylococcal strains (1 clinical isolate and five reference strains). They analyzed peaks between 500 and 10,000 m/z. With this approach they identified 2 MSSA specific peaks (2,548 and 2,647 m/z) and 5 MRSA specific peaks (581, 1,140, 1,165, 1,229, and 2,127 m/z). However, the authors did not verify their findings with a prospective evaluation of clinical samples.

Two years later Du et al. (2002) analyzed 76 clinical *S. aureus* strains. They studied a mass range of 600–3,500 m/z. With this method only 74% of the above mentioned 76 *S. aureus* strains could be identified as *S. aureus*. However, clustering of the spectra revealed two main clusters with a good correlation to susceptibility or resistance, i.e., MSSA or MRSA. But concordance was not 100%. 7 strains, which were *mecA* negative (PCR) clustered into the MRSA group. Unfortunately the authors did not trace back the differences between MSSA and MRSA spectra to the individual peaks but published two “typical” spectra of MSSA and MRSA. A close look at these spectra shows that one of the different peaks is the peak at 2,413 m/z, which we now know is related to PSM-mec peptide (Josten et al., 2014).

Again in 2002 Bernardo and colleagues (Bernardo et al., 2002) studied two well-characterized reference strains, ATCC 29213 (MSSA) and ATCC 43300 (MRSA), and compared their spectra to clinical isolates. They studied a mass range of 1,000–10,000 m/z and in contrast to all other studies mentioned in this review they used the reflector mode not the linear mode of the MALDI-TOF MS. They could not find an MRSA-specific fingerprint.

Ten years later in 2012 the group of S. Gatermann (Szabados et al., 2012) studied a pair of isogenic *S. aureus* strains, which harbored or lacked a certain SCCmec (staphylococcal chromosome *mec*). They did not find evidence for a characteristic spectrum or peak associated with methicillin-resistance. Unfortunately the authors neither mentioned the SCC cassette type present in the resistant strain nor published a peak list for further reference.

**Figure 1:**
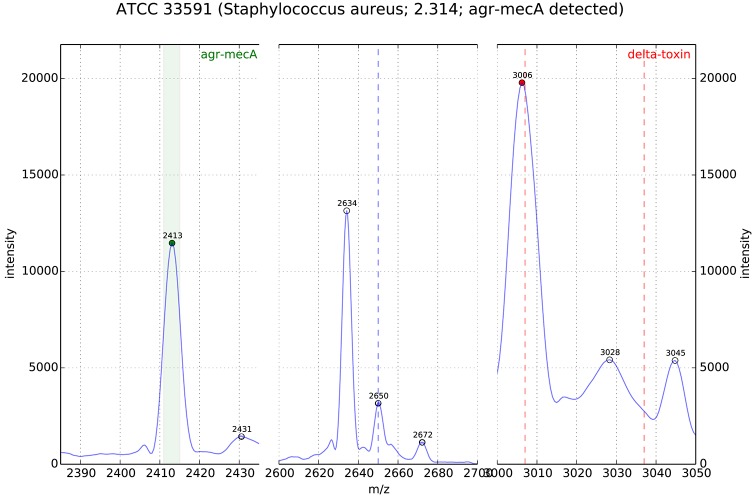
MALDI-TOF MS of *S. aureus* (ATCC33591, MRSA); the peak analysis shows the PSM-mec related peak in the detection window m/z 2411-15 (origin: DGHM 2016, poster 139, A. Pranada and co-workers “Optimization and Evaluation of MRSA Detection by Peak Analysis of MALDI-TOF Mass Spectra”).

In Josten et al. (2014) screened a very diverse collection of 220 MRSA strains for the presence of a special peak at 2415 m/z. Using the RNA antisense technology they first established that the peak at 2,415 m/z in a *S. aureus* spectrum was correlated with the expression of PSM-mec. PSM-mec is a small peptide, which is encoded on three different SCCmec cassette types (i.e., type II, III, and VIII). Because PSMmec is encoded on the SCCmec cassette its expression is associated with a methicillin resistant phenotype in *S. aureus*. Afterwards they prepared spectra of 220 strains and visually checked for the presence of a peak at 2,415 m/z. Their conclusion was that as soon as the peak was discernible in a spectrum the strain most probably was an MRSA. However, during analysis they had to realize that sample preparation was extremely important for the detection of the peak. As soon as the background of the measurement was high it was difficult to identify the peak. In Pranada et al. (2016) used prototype software for automated detection of a peak between 2,411 and 2,415 m/z in *S. aureus* spectra generated during routine identification. They reevaluated 1304 spectra of *S. aureus* isolates from their microbiology routine, which had been acquired from 2011 to 2014. For each of these isolates the information on methicillin resistance was available (MICs determined using VITEK2, bioMerieux). Of 211 MRSA strains 49 (23.2%) had a peak at 2,411–2,415 m/z. Importantly none of the 1093 MSSA strains had a peak at 2,411–2,415 m/z, which makes this peak extremely specific for MRSA (see Figure 1).

During ECCMID 2017 confirmatory data were presented with a positivity rate for PSM-mec of 15.7% (Timke et al., 2017).

### Vancomycin-resistance: VRE–vanA and vanB

However, MRSA was not the only focus of work for rapid susceptibility testing. From a clinical perspective vancomycin-resistant enterococci (*vanA* or *vanB* positive) are in the focus of infectious disease specialists, too. In 2012 Griffin and co-workers (Griffin et al., 2012) published a paper on the discrimination between *vanB* and *non-vanB* carrying enterococci in Australia using MALDI-TOF. The group had collected 67 consecutive vancomycin resistant *Enterococcus faecium* isolates (*vanB* postive, confirmed by PCR) from the beginning of January 2009 to the end of June 2010. Among the controls were 8 strains, which were phenotypically resistant to vancomycin but were *vanB* and *vanA* negative by PCR. The mechanism of non-susceptibility to vancomycin of the latter strains was not investigated. Five differentiating peaks were identified using the program ClinPro Tools version 2.0 (2,211, 4,717, 5,095, 5,946, and 8,328 m/z). For a prospective validation of these findings reference spectra of *vanB* positive and negative strains were added to the database. Phenotypically vancomycin resistant enterococci not carrying the *vanB* gene were designated as VRE negative in the database. Unfortunately the total number of added spectra was not mentioned in the original publication. From January 2012 to February 2012 strains from 129 patient samples were used for validation. Altogether 281 different colonies were tested. Compared to phenotypic methods 97.5% were correctly identified and 2.5% were incorrectly identified, 1.1% were false positive and 1.4% false negative.

Two years later the group of Nakano (Nakano et al., 2014) published a paper on the discrimination between *vanA*-positive and *vanA*-negative *Enterococcus faecium*. They studied 61 *vanA*-positive strains from a surveillance program (rectal swabs and stool, 2005–2013) and 71 *vanA*-negative strains from blood cultures (2009–2013). All but two strains were isolated from patients from the Kyoto region in Japan during the mentioned time periods. Using classification models (genetic algorithm, supervised neural network, QuickClassifier) revealed five peaks differentiating between the two entities: 3,184, 5,702, 7,415, 7,445, and 12,662 m/z. Unfortunately the authors neither published typical spectra nor further elaborated which peak was characteristic for which genotype. Additionally they did not try to clear up the identity of the peak, i.e., which peptide or protein caused the peak. This is especially unfortunate because all strains stem from one region. The discriminating peaks might be an artifact due to clonality.

### Carbapenem-resistance: MRGN and *B. fragilis*

In Lau et al. (2014) studied 38 isolates of *Klebsiella pneumoniae*, which carried the pKpQIL plasmid. This plasmid contains the *bla*_KPC_ gene, which encodes a carbapenemase called KPC (*K. pneumoniae* carbapenemase). KPC causes high carbapenem resistance. They started by comparing spectra from *bla*_KPC_ positive and negative strains as determined by PCR. They worked with 19 *bla*_KPC_ positive isolates (ATCC BAA-1705 and 18 clinical isolates from an outbreak in 2011) and 19 *bla*_KPC_ negative isolates (ATCC BAA-1706 and 18 clinical isolates). Visual comparison of the spectra revealed a peak at 11.109 m/z, which was only present in the *bla*_KPC_ positive isolates. Using transformation and additional proteomic methods they could confirm the identity of the protein causing the peak. Most importantly for laboratory routine they could show that this peak can be detected directly from blood-cultures, which had been artificially inoculated with two different *bla*_KPC_ positive isolates. In 2016 the group of Paolo Gaibani in Bolognia tested this assay with 34 KPC-producing *K. pneumoniae* strains of which 30 (88.2%) were positive for the 11.109 m/z peak. Further genetic analysis revealed that the 4 strains negative for the 11.109 m/z peak could be explained by different isoforms of Tn*4401*. Only TN*4401a* is commonly associated with the 11.109 m/z peak (Gaibani et al., 2016). In 2018 the same group published a study on 140 well-characterized *K. pneumoniae* strains collected between 2011 and 2017 and found an overall accuracy of 98%, a positive predictive value of 98% and a negative predictive value of 97% (Gaibani et al., 2018).

In Nagy et al. (2011) from Hungary described the separation of *Bacteroides fragilis* strains into two divisions using MALDI-TOF MS spectra: division I (all strains were *cfiA*-negative) and division II (all strains were *cfiA*-positive). Clinically these divisions are of interest because the presence of the *cfiA*-gene is associated with carbapenem resistance in *B. fragilis*. The study included 38 different clinical *B. fragilis* strains with known *cfiA* gene status (determined by PCR) and two reference strains: NCTC 9343 (*cfiA* negative) and TAL 3636 (*cfiA* positive). The clinical strains originated from Europe and the US. Especially in the mass range between 4,000 and 5,500 m/z they could identify characteristic differences in the respective spectra using ClinPro Tools v. 2.2. Altogether they identified 20 peaks, which differentiated between divisions I and II (division I (*cfiA*-negative): 4,711, 4,817, 5,017, 5,204, 5,268, 7,292, 9,421, 9,631, 10,404, and 10,530 m/z; division II (*cfiA*-positive): 4,688, 4,826, 5,002, 5,189, 5,282, 7,321, 9,375, 9,649, 10,374, and 10,558 m/z). Unfortunately the identities of the peaks were not studied. Therefore, we do not know whether at least one of the peaks is resistance mechanism correlated or whether the peak differences are due to clonality. A further analysis of spectra from 277 clinical samples previously acquired showed that some of these peaks could be found in spectra from clinical routine. They correlated 100% with PCR positivity. Finally the authors generated a specialized database consisting of 2 MSPs only (*cfiA* +, *cfiA-*) and challenged it with spectra from 9 *cfiA*-positive and 19 *cfiA*-negative strains. Matching was correct in 100%. These findings were confirmed in 2011 and 2014. A group from Belgium studied 248 isolates of *B. fragilis* collected between 2003 and 2011 with very good differentiation between *cfiA*-positive and negative strains (Wybo et al., 2011) and in 2014 Nagy and co-workers confirmed their original findings with 60 *B. fragilis* isolates from polymicrobial or severe infections (Fenyvesi et al., 2014). From a laboratory perspective the differentiation between divisions II and I is interesting, because until today only the division II strains harbor the *cfiA* gene. This gene does not correlate 100% with carbapenem resistance. However, it can serve as an indicator for the necessity to perform further susceptibility testing. Only *cfiA* positive isolates showed imipenem MICs >4 mg/L (EUCAST cut-off for imipenem resistance). On the other hand from the data presented it is justified to call division I strains susceptible to carbapenems without further susceptibility testing. But if this workflow is implemented one should be aware of the obvious risk that other carbapenem resistance mechanisms are overlooked.

All of the described methods have the same big advantage for the clinical routine. All of these peaks can be identified in the spectra, which are generated during the normal identification workflow. No additional assay is necessary; no additional incubation time is needed. The peaks can be detected visually or by software tools, which already exist but are not routinely available yet. However, all of the papers (except two) share the same incompleteness. They do not correlate the peaks with the peptides or proteins they represent. Therefore, it cannot be excluded that these peaks are discriminatory only in the strain collections used and are an artifact from clonality rather than truly discriminatory worldwide.

### Assays using alterations of antibiotics as read-out (hydrolysis, decarboxylation, acetylation)

The most diverse class of antibiotics is the class of the beta-lactams. This class contains penicillins, cephalosporins, and carbapenems. Their spectrum of antibacterial activity is versatile; however, they share a common feature. They can be inactivated by hydrolysis. In the clinical setting this hydrolysis is caused by enzymes produced by bacteria. Thousands of these bacterial enzymes (i.e., beta-lactamases) have been described and their number is still rising. The mechanism of hydrolysis is always exactly the same. The enzymes can break the beta-lactam ring of the beta-lactams open and a single H_2_O is linked to the molecule. Depending on the buffer the resulting hydrolysate is unstable and a spontaneous decarboxylation can take place. In terms of susceptibility testing this degradation process can be monitored with MALDI-TOF. The addition of water increases the original antibiotic mass by 18 Da and the decarboxylation decreases the molecular mass by 44 Da. Taken together this results in an absolute loss of 26 Da (44–18 Da) compared to the original mass of the antibiotic. To be able to visualize this degradation a bacterial suspension with the antibiotic in question has to be prepared and incubated for various amounts of time.

**Figure 2:**
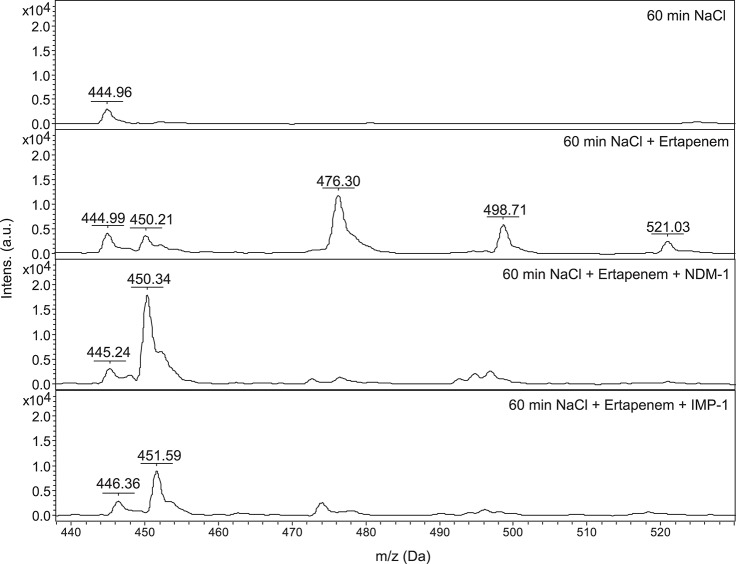
Ertapenem degradation: NDM-1 carrying *K. pneumoniae*, IMP-1 carrying *P. aeruginosa*, 60 min of incubation at 36°C, NaCl: 0.45%, ertapenem concentration: 0.5 g/l, x-axis: mass per charge in Dalton (m/z, Da), y-axis: intensity: arbitrary units. Data are representative of more than three independent experiments. JCM, 2011, 49, 3321–3324, doi: 10.1128/JCM.00287-11, original Figure 1, reproduced with permission from American Society for Microbiology.

In 2011 two European groups published this phenomenon simultaneously for the detection of carbapenem degrading enzymes (i.e., carbapenemases). The group of Burckhardt and Zimmermann (2011) studied 47 clinical isolates carrying different carbapenemases (KPC, NDM, IMP, VIM) and ertapenem resistance (MIC ≥4 mg/L). They included 30 clinical strains carrying other resistance mechanisms (ESBL, K1), which did not cause ertapenem resistance. They studied a mass range of 440–530 m/z. A spectrum of ertapenem as it is used for patient therapy showed 4 peaks: 476 m/z (ertapenem without sodium), 498 m/z (monosodium salt), 521 m/z (disodium salt) and 450 m/z (hydrolyzed and decarboxylated ertapenem) (see Figure 2). Depending on the enzyme the mixtures of bacteria and ertapenem had to be cultivated between 1 and 2.5 h. The read-out they used was total disappearance of the ertapenem peaks at 476, 498, and 521 m/z. Discrimination between carbapenemase carrying and non-carrying strains was 100%. In this proof-of-principle study the authors used a reaction volume of 1 ml, a 10 μl loop full of bacteria, a concentration of 0.5 g/L of ertapenem in 0.9% NaCl and α-cyano-4-hydroxycinnamic acid as matrix.

At the same time the group of Hrabák et al. (2011) studied the degradation of meropenem. They used 124 different strains of which 30 carried carbapenemases (KPC, NDM, IMP, and VIM). All the other strains were controls carrying other resistance mechanisms resulting in elevated carbapenem MICs (55 strains) or were completely susceptible to meropenem and imipenem (39 strains). They looked at masses from 360 to 600 m/z. The spectrum of meropenem showed three characteristic peaks: 383 m/z (meropenem without sodium), 405 and 427 m/z (sodium salts). After hydrolysis the following peaks were discernible: 401, 423, 445, and 468 m/z (sodium salts). They used 1 ml of a McFarland (McF) 8 bacterial solution, a 0.1 mM meropenem solution in 20 mM Tris-HCl (pH 6.8) and incubated in a final reaction volume of 50 μl for 3 h. Of the three different matrices used (α-cyano-4-hydroxycinnamic acid, 2,5-dihydroxybenzoid acid, DHB and 2,5-dihydroxyacetophenone, DHAP) DHB worked best under the described circumstances. Read out was the disappearance of at least one of the following peaks: 383 or 405 m/z. Their analysis of the strains using the above mentioned workflow produced one false negative and two false-positive results. In 2015 the same group published a modification of their initial method (Papagiannitsis et al., 2015). Addition of 50 mM NH_4_HCO_3_ (pH 7.0) to the original reaction mix improved detection of OXA-48 producing strains from 3 of 19 strains to 19 of 19 strains. In 2017 the same group compared meropenem and imipenem for the detection of carbapenemases. The imipenem assay achieved a higher sensitivity (97%) and specificity (100%) for the testing of *P. aeruginosa* (250 strains tested), whereas the meropenem assay achieved a higher sensitivity (99%) and specificity (100%) for Enterobacteriaceae (124 strains tested) (Rotova et al., 2017). In 2017 another group published results for a slightly modified meropenem assay testing 1185 enterobacterial strains from Italy, which carried mainly KPC or VIM enzymes (Calderaro et al., 2017). It showed that the integrity of meropenem is an important factor in the analysis of the read-out and the mere presence and absence of meropenem specific peaks is not suitable as the only read-out.

In Sparbier et al. (2012) published another version of the beta-lactamase hydrolysis assay. It was extended to ampicillin, piperacillin, ceftazidime, cefotaxime and imipenem and results for ertapenem and meropenem were confirmed. In this study the authors used a reaction volume of 10 μl, a 1 μl loop full of bacteria and an incubation time of up to 3 h. Concentrations were different for all substances: ampicillin (10 mg/ml), piperacillin (1 mg/ml), ceftazidime (0.25 mg/ml), cefotaxime (0.5 mg/ml), imipenem (0.5 mg/ml), ertapenem (0.5 mg/ml), meropenem (0.5 mg/ml). They studied a mass range between 290 and 600 m/z. Only 10 different strains were used in this study including *E. coli* DH5α and 9 different clinical isolates carrying different beta-lactamases (AmpC, ESBL and KPC). But they were the first to use beta-lactamase inhibitors for inhibition of beta-lactamases in this kind of assay. Interpretation of data was done visually and the assay was considered positive for the presence of a beta-lactamase if the intensities of the peaks of the hydrolyzed forms represented 80% or more of the intensities of the non-hydrolyzed plus the hydrolyzed forms of the respective antibiotic. In 2014 Jung and co-authors confirmed that the assay works for 3rd-generation cephalosporins/Enterobacteriaceae and aminopenicillins/*E. coli* directly from blood culture (Jung et al., 2014b). In 2016 it was shown that the hydrolysis assay (imipenem only) works directly from positive blood cultures for Enterobacteriaceae, *P. aeruginosa* and *A. baumannii* (Oviaño et al., 2016) In 2018 another group successfully used the hydrolysis assay on blood cultures with ampicillin, piperacillin, cefotaxime, ceftazidime and meropenem (Lee et al., 2018). Interestingly the hydrolysis assay seems to be influenced by the agar type used for cultivation of strains. Strains grown on MacConkey agar tended to give false negative results in a study in 2016 (Ramos et al., 2016). This effect was mainly observed for *A. baumannii*.

In 2014 the ESCMID Study Group on Anaerobic Infections confirmed that the ertapenem hydrolysis assay worked with *cfiA* positive *B. fragilis* strains, too (Johansson et al., 2014a). Johansson and co-workers studied a group of 28 different *B. fragilis* strains of which 16 carried the *cfiA* gene and had different levels of elevated ertapenem MICs (≥2 mg/L). These elevated ertapenem MICs correlated in 10/16 cases with elevated imipenem MICs (≥2 mg/L). They used the pellet of 1.5 ml McF 4 bacterial solutions and 20 μl of a 10 mM ammonium hydrogen citrate buffer for incubation with 0.5 mg/mL of ertapenem. All of the 16 *cfiA* positive strains hydrolyzed ertapenem after 2.5 h of incubation. This hydrolysis could be blocked by 2,6-Pyridinecarboxylic acid (DPA), a metallo-beta-lactamase inhibitor. All 12 *cfiA* negative strains did not show any hydrolysis of ertapenem. The same year the same group could show that the assay also worked with pellets from positive blood culture bottles despite the presence of blood components (Johansson et al., 2014b).

Beta-lactams are not the only antibiotics, which can be inactivated by enzymes. The plasmid-encoded acetyltransferase AAC(6‘)-Ib-cr inactivates quinolones via acetylation. In 2016 Pardo and co-workers (Pardo et al., 2016) described a MALDI-TOF MS based method to detect acetylation of norfloxacin. Acetylation increases the mass of the acetylated substance by 42 Da. They studied a collection of 113 ESBL-producing Enterobacteriaceae of French origin. 102 of these 113 strains were phenotypically resistant to norfloxacin, however, only 64 strains carried the *aac(6*′*)-IB-cr* gene. They used a 1 μl loop full of bacteria and incubated them in 10 μl of a 0.03 or 0.5 mg/mL norfloxacin solution for 4 h at 35°C. They studied the mass region between 270 and 420 m/z. Norfloxacin produced peaks at 320 m/z (norfloxacin without sodium) and 342 m/z (mono sodium salt). Acetylation should increase the weight of the respective substance by 42 Da. And after incubation of norfloxacin with an AAC(6′)-Ib-cr producing strain peaks at 362 m/z (acetylated norfloxacin) and 384 m/z (acetylated mono sodium norfloxacin) could be seen in the respective spectra. As read-out they calculated the areas under the curve (AUCs) for the respective peaks. Optimal cut-offs for positivity were determined using receiver operating characteristics (ROC) curve analysis. The lower concentration of norfloxacin (0.03 mg/mL) seemed to be better suited for the analysis. They authors claimed that their assay had a sensitivity of 98% and a specificity of 100% for the detection of AAC(6‘)-Ib-cr.

### Assays using peak shift as read-out (incorporation of ^13^C)

In February 2013 Demirev and co-workers described a very universal method to determine susceptibility in bacteria (Demirev et al., 2013). They used two different culture media to grow bacteria. The two media differed by their carbon component. One medium contained ^12^C, in the other medium 98% of all carbon atoms were ^13^C, i.e., heavier. The idea was to monitor whether the bacterium was still capable of growing in the presence of the antibiotic or not. This could be deduced from the spectrum generated from bacteria grown in a solution containing ^13^C and antibiotic. If the bacterium was resistant it could grow in the presence of the antibiotic and would incorporate ^13^C and the spectrum would shift to higher m/z. In theory this principle could be applied to all antibiotics and should work irrespective of resistance mechanisms.

**Figure 3:**
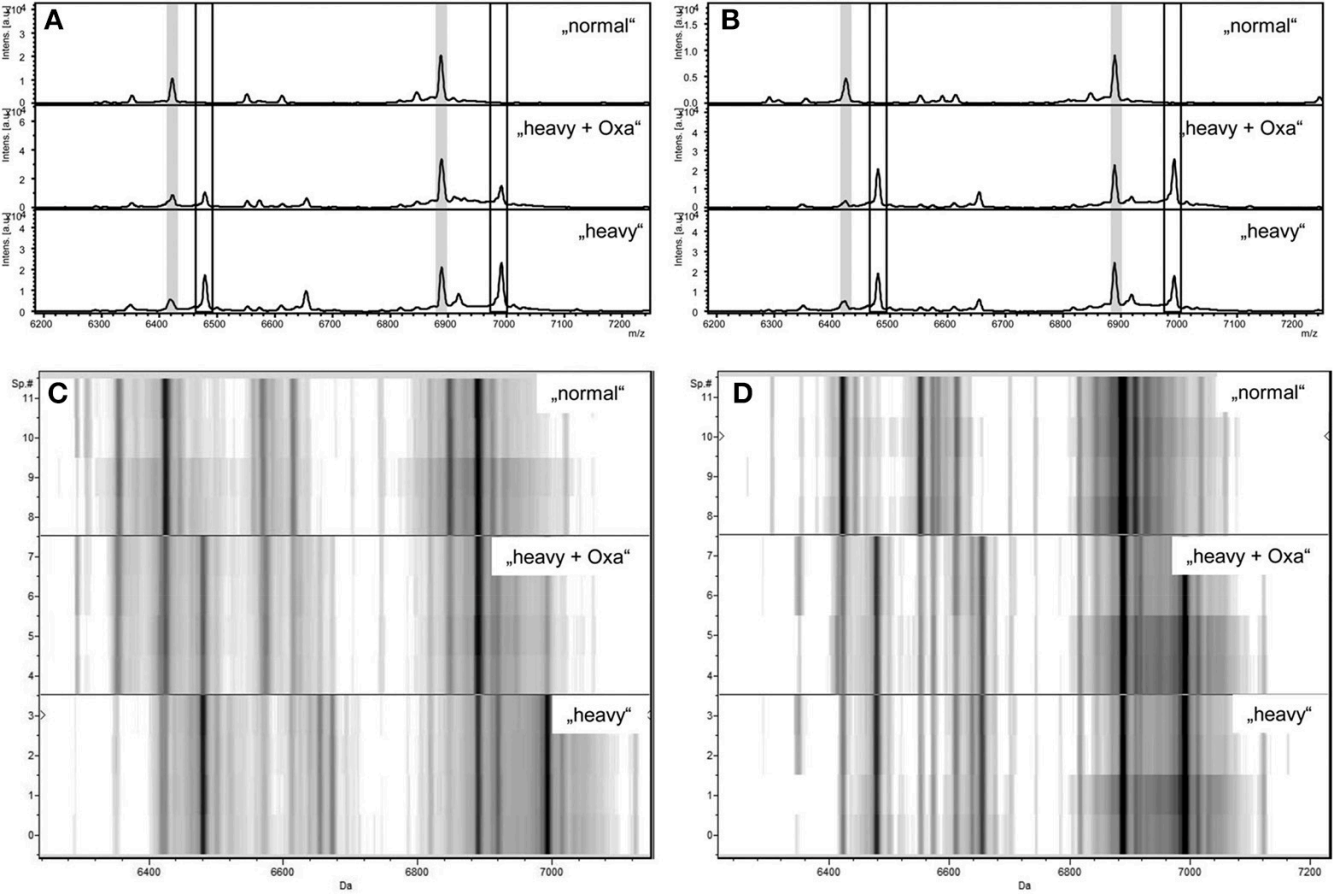
Zoom of MALDI-TOF MS spectra displayed in flexAnalysis **(A,B)** and ClinProTools **(C,D)** in the mass range between 6.200 and 7.200 Da of a susceptible *S. aureus* strain **(A,C)** and a resistant *S. aureus* strain **(B,D)** after incubation with normal lysine (“normal”), with “heavy” lysine (“heavy”), or with “heavy” lysine and oxacillin (“heavy + Oxa”). Peaks correspondig to “normal” proteins are highlighted in light gray **(A,B)**. Peaks corresponding to “heavy” proteins are indicated by the boxes **(A,B)**. y axes give the numbers of multiple measurements **(C,D)**. Intes. [a.u.], intensity [arbitrary units]; Sp.#, spectrum number. JCM, 2013, 51, 3741–3748, doi: 10.1128/JCM.01536-13, original Figure 1, reproduced with permission from American Society for Microbiology.

In August 2013 Sparbier and colleagues published data created with *S. aureus* and media either containing ^12^C or ^13^C-labeled lysine (Sparbier et al., 2013). The authors called this assay MBT-RESIST (MALDI Biotyper resistance test with stable isotope-labeled amino acids). They started with 10 MSSA and 10 MRSA strains and further evaluated their findings with 28 *S. aureus* strains from patient samples. As antibiotics they used oxacillin (60 mg/L) or cefoxitin (40 mg/L). Bacteria were incubated at 37°C for 3 h in a volume of 100 μl and a final concentration of 3.5 × 10^6^ cells/mL. Each test consisted of three tubes. Tube 1 contained ^12^C medium and no antibiotic. Tube 2 contained ^13^C medium and no antibiotic. Tube 3 contained ^13^C medium *and* antibiotic. Tube 1 and 2 served as controls. The spectrum created from tube 3 decided whether a strain was rated susceptible or resistant against the respective antibiotic (see Figure 3).

The spectrum of a resistant strain in tube 3 would resemble more the spectrum from tube 2 than from tube 1. The spectrum of a susceptible strain in tube 3 would resemble more the spectrum from tube 1 than from tube 2. However, depending on the strain studied the acquired spectra were ambiguous. Therefore, the authors decided to use automated spectra analysis for the interpretation of the test. With this workflow one susceptible strain was falsely considered to be resistant using the oxacillin set-up. Three strains were wrongly classified using the cefoxitin set-up. Two main factors were responsible for false classification; a) the quality of the spectra; a noisy background led to false classifications; b) growth kinetics of strains; strains growing slowly were difficult to correctly classify after only 3 h of incubation.

In November 2013 the same group published that this approach works not only for *S. aureus* and oxacillin/cefoxitin but also for *Pseudomonas aeruginosa* and meropenem, tobramycin and ciprofloxacin (Jung et al., 2014a). In contrast to their initial publication they used a reaction volume of 300 μl and looked at a mass range of 2,000–1,0000 m/z. The authors used 10 strains of *P. aeruginosa* to establish their workflow and validation sets of 30 strains for each antibiotic (15 susceptible and 15 resistant strains each). To ease the interpretation of the spectra meropenem had to be added 30 min before the ^13^C lysine to the reaction tube. According to the authors even strains with MIC values close to the breakpoint were classified correctly. Unfortunately neither sensitivity/specificity nor positive/negative predictive values were explicitly mentioned in the publication.

### Assays using quantification of the area under the curve as read-out

**Figure 4:**
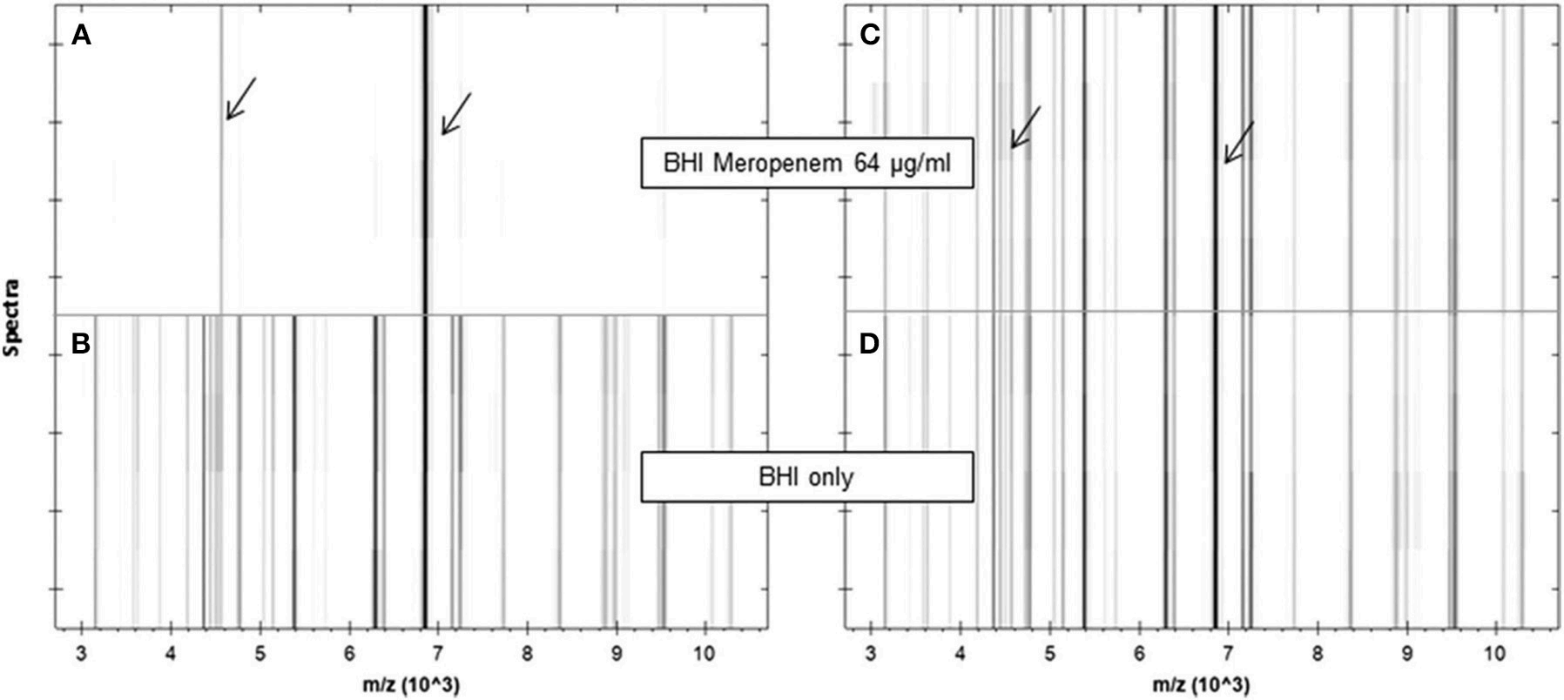
Pseudogel views of the mass range between 3 and 10 kDa of a susceptible **(A,B)** and a resistant **(C,D)** *K. pneumoniae* strain after incubation in the absence (lower panels) or presence (upper panels) of meropenem (64 μg/ml) for 1 h. For each incubation, four spectra acquired from two different spots are shown. Internal standard peaks are marked by arrows. JCM, 2014, 52, 4155–4162, doi: 10.1128/JCM.01872-14, original Figure 1, reproduced with permission from American Society for Microbiology.

In Lange et al. (2014) published a completely new MALDI-TOF MS method for susceptibility testing of bacteria. The authors used the abbreviation MBT-ASTRA (MALDI Biotyper antibiotic susceptibility test rapid assay) for this test. They determined the susceptibility of 108 *Klebsiella* spp. isolates (see Figure 4) against meropenem using relative growth. Each test consisted of two tubes. Tube 1 contained meropenem and tube 2 did not contain meropenem. They used meropenem at a concentration of 8 mg/L, a reaction volume of 200 μl (BHI, 0.5 McFarland solution) and an incubation time of 1 h. The resulting spectra were normalized to the maximum peak and the resulting spectrum subdivided into 100 equally spaced thresholds (relative intensity range). The number of peaks above each threshold was counted and plotted against the threshold. The area under this curve, not under the initial spectrum (AUC) was determined for each measurement. Finally the relative growth was calculated as follows: AUC (+meropenem)/AUC (–meropenem). A relative growth of >0.4 was indicative of resistance to meropenem. This protocol was tested against 94 *K. pneumoniae* and 14 *K. oxytoca* strains and gave five false positive and one false negative result. All other 102 results were correct. The strain, which gave the false negative result, was a strain expressing heterogeneous resistance. An explanation for the false positive results could not be given. A repetition of the assay for these five strains gave the correct results. Additionally this assay worked with artificially inoculated blood culture bottles. 17 of 18 *Klebsiella* sp. were correctly classified.

Two years later Jung and co-workers published a slightly changed version of the MBT-ASTRA for gentamicin, ciprofloxacin, cefotaxime and piperacillin-tazobactam (Jung et al., 2016). In contrast to the first description of the assay they used 200 μl of liquid Mueller-Hinton broth (OD_600_ of 0.007; 5 × 10^6^ cfu/mL) instead of BHI broth and incubated for up to 3 h. First they spiked 30 blood culture bottles (BD BACTEC Plus Aerobic/F and Anaerobic) with different Enterobacteriaceae (*E. coli, Enterobacter* spp., *Klebsiella* spp. and *P. mirabilis*) and used gentamicin (4 mg/L) and ciprofloxacin (1 mg/L) as antibiotics. In a second step they tested 99 real-time patient blood-cultures (mainly *E. coli* and *Klebsiella* spp.) for non-susceptibility to ciprofloxacin (1 mg/L), cefotaxime (2 mg/L) and piperacillin-tazobactam (16/4 mg/L). Both parts of the study showed that all strains, which were fully susceptible or fully resistant to any of the tested antibiotics were accurately classified. Problems with correct classification were seen in case of poor growth in the tube without antibiotic, too short incubation times and with strains with MICs near the antibiotic concentration used in the assay.

In 2018 a first study of *B. fragilis* and MBT-ASTRA was published (Justesen et al., 2018). In this proof of principle study the authors demonstrated the suitability of MBT-ASTRA for the susceptibility prediction for clindamycin, meropenem and metronidazole.

### New developments

**Figure 5:**
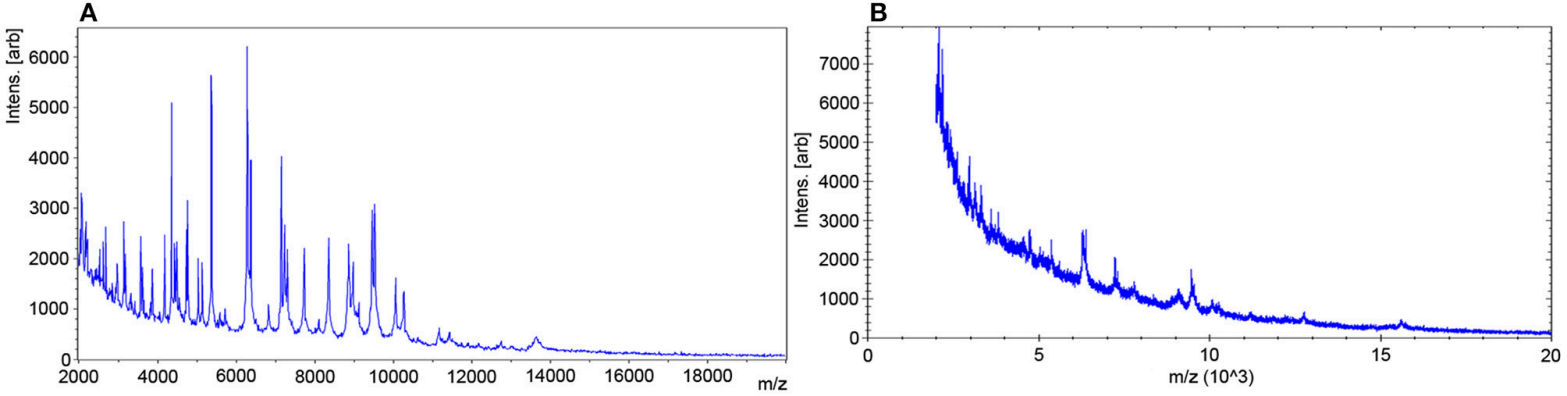
Evaluation of the MALDI-TOF MS data **(A)** Meropenem-resistant isolate, MALDI Biotyper finding: *K. pneumoniae*. **(B)** Meropenem-susceptible isolate, MALDI Biotyper finding: No identification. With permission from Idelevich, E. A., Sparbier, K., Kostrzewa, M., and Becker, K. Rapid detection of antibiotic resistance by MALDI-TOF mass spectrometry using a novel direct-on-target microdroplet growth assay. Clin. Microbiol. Infect. 24, 738–743, Elsevier, © 2018.

In 2017 the direct-on-target microdroplet growth assay was described (Idelevich et al., 2018). In this proof-of-principle study Idelevich and co-workers studied meropenem susceptible and resistant strains of *K. pneumoniae* and *P. aeruginosa* (12 susceptible and 12 resistant strains of each species). Bacterial suspensions with or without 2 mg/L of meropenem were applied to a target and incubated for up to 18 h on the target. The subsequent MALDI-TOF MS analysis showed a successful identification for meropenem-resistant isolates only. Meropenem-susceptible strains showed spectra with the result “no identification” (see Figure 5).

### What can we hope for in the future?

From a patient‘s perspective susceptibility testing should be accurate and rapidly available. Under ideal circumstances the time to report for rapid susceptibility testing would be a few minutes and availability of test results would be 24/7. Accuracy would mean that the susceptible/intermediate/resistant (SIR) result had a 100% positive predictive value for successful therapy, i.e., if an antibiotic is classified susceptible the patient should be successfully treated with it in 100 of 100 cases.

In reality time to report for susceptibility testing is >18 h for agardiffusion and >6–24 h for MIC determination after successful cultivation of the bacterium. In general test results are available during daytime and generating large data for positive predictive values of SIR determination is hardly feasible with patients.

The fastest possible susceptibility testing with MALDI-TOF MS is the simultaneous detection of one or more characteristic “resistance” or “susceptibility” peaks in the spectra generated for identification of the respective strain. Automated detection of these peaks is already feasible but what is largely missing is the identification of the protein behind this/these peak(s). This set-up would help very much during clinical routine in cases where for one particular bacterium the susceptibility to one single antibiotic is needed. Ideally the peak is caused by a protein causing resistance or at least correlated to the dominant resistance mechanism (e.g., MRSA and PSM-mec).

However, what infectious disease specialists wish for are rapid and reliable antibiograms displaying results for >10 antibiotics comparable to what is available today with MIC determination. The degradation method could be part of this set-up if the resistance mechanism to the antibiotic tested is enzymatic degradation. However, MBT-RESIST and/or MBT-ASTRA seem to be more suitable to achieve that goal. They are able to detect non-susceptibility due to different resistance mechanisms (e.g., efflux, target modification and degradation). With the lack of new antibiotic substances for therapy the ID specialist must optimize therapy by using the MIC values. With the current MIC assays it is possible to report the MIC values and use them for therapy optimization. Nothing comparable to an MIC was published for bacteria and susceptibility testing with MALDI-TOF MS. All assays use a single defined concentration of an antibiotic. However, for *Candida albicans* Marinach and co-workers published the concept of the minimal profile change concentration (MPCC) (Marinach et al., 2009). Instead of using a single concentration of fluconazole they performed a serial dilution from 128 to 0.125 μg/mL. The MPCC was defined as the lowest drug concentration at which a mass spectrum profile change was detected. This concept was further evaluated with 16 strains of *C. albicans*. MICs had been determined following the CLSI guidelines. MPCC and MIC results were highly correlated (94–100%). An essential prerequisite for the success of these assays is their automation, miniaturization and standardization to make large-scale studies possible. Aspects that still have to be addressed during these studies are inoculums, time of incubation, concentration(s) of antibiotics, optimal matrices, background reduction and automated interpretation of results. Hopefully cost for all of these necessary developments will be manageable and the final test will prove to be cost effective. Cost will be an important factor determining or even deciding whether MALDI-TOF MS based susceptibility testing will be the upcoming technique for susceptibility testing. The main competitor for MALDI-TOF MS for susceptibility testing currently is the detection of known resistance determinants using PCR, that is genome based assays not proteome based assays. Even whole genome sequencing for “susceptibility testing” is under discussion. Very recently Greninger published an excellent review covering the pros and cons, the benefits and pitfalls of diagnostic metagenomics (Greninger, 2018) and therefore this subject is not further discussed here.

During the ongoing evaluation of MALDI-TOF MS for susceptibility testing it will be necessary to reach a broad consensus on two important aspects of susceptibility testing with MALDI-TOF MS. First we need to account for resistance mechanisms, which take time to take action. For example some enzymes are very slow. If incubation times are too short the susceptible/resistant classification will be wrong. For these mechanisms we need reaction conditions with which they can be detected within a few hours. Second, we need to agree on whether correlation of MALDI-TOF MS susceptibility results to MIC values is enough or whether we need *in vivo* therapy studies. Today we observe cases of therapeutic failure despite susceptible MIC that is *in vitro* susceptibility. One possible reason for therapeutic failure is that the initial *in vitro* susceptibility testing result does not mirror the *in vivo* activity of the antibiotic. Especially for strains with a divergent MALDI-TOF MS and MIC classification it would be interesting to determine the *in vivo* action, *in vivo* veritas. The breakthrough for antibiotic susceptibility testing with MALDI-TOF MS would be to demonstrate that results from MALDI-TOF MS susceptibility testing correlate better with successful therapy than results from traditional susceptibility testing (agardiffusion, E-Test, MIC-determination using automated systems).

If we find good solutions for these aspects the MALDI-TOF MS technology undoubtedly is the most promising type of assay for rapid and reliable susceptibility testing within the next 10 years.

## Author contributions

IB drafting of manuscript, writing of manuscript, revision of manuscript. SZ revision of manuscript.

### Conflict of interest statement

The authors declare that the research was conducted in the absence of any commercial or financial relationships that could be construed as a potential conflict of interest.
